# Risk factors of hemorrhagic transformation in acute ischaemic stroke: A systematic review and meta-analysis

**DOI:** 10.3389/fneur.2023.1079205

**Published:** 2023-02-20

**Authors:** Jiacheng Sun, Christina Lam, Lauren Christie, Christopher Blair, Xingjuan Li, Freda Werdiger, Qing Yang, Andrew Bivard, Longting Lin, Mark Parsons

**Affiliations:** ^1^Sydney Brain Centre, The Ingham Institute for Applied Medical Research, Liverpool, NSW, Australia; ^2^South Western Sydney Clinical School, University of New South Wales, Sydney, NSW, Australia; ^3^Melbourne Brain Centre at Royal Melbourne Hospital, Melbourne, VIC, Australia; ^4^Department of Medicine, University of Melbourne, Melbourne, VIC, Australia; ^5^Allied Health Research Unit, St Vincent's Health Network Sydney, Sydney, NSW, Australia; ^6^Faculty of Health Sciences, Australian Catholic University, North Sydney, NSW, Australia; ^7^Department of Neurology and Neurophysiology, Liverpool Hospital, Sydney, NSW, Australia; ^8^Queensland Department of Agriculture and Fisheries, Brisbane, QLD, Australia; ^9^Apollo Medical Imaging Technology Pty Ltd., Melbourne, VIC, Australia

**Keywords:** stroke, risk factor, intracranial hemorrhage, hemorrhagic transformation, reperfusion therapy, intravenous thrombolysis, endovascular thrombectomy

## Abstract

**Background:**

Hemorrhagic transformation (HT) following reperfusion therapies for acute ischaemic stroke often predicts a poor prognosis. This systematic review and meta-analysis aims to identify risk factors for HT, and how these vary with hyperacute treatment [intravenous thrombolysis (IVT) and endovascular thrombectomy (EVT)].

**Methods:**

Electronic databases PubMed and EMBASE were used to search relevant studies. Pooled odds ratio (OR) with 95% confidence interval (CI) were estimated.

**Results:**

A total of 120 studies were included. Atrial fibrillation and NIHSS score were common predictors for any intracerebral hemorrhage (ICH) after reperfusion therapies (both IVT and EVT), while a hyperdense artery sign (OR = 2.605, 95% CI 1.212–5.599, *I*^2^ = 0.0%) and number of thrombectomy passes (OR = 1.151, 95% CI 1.041–1.272, *I*^2^ = 54.3%) were predictors of any ICH after IVT and EVT, respectively. Common predictors for symptomatic ICH (sICH) after reperfusion therapies were age and serum glucose level. Atrial fibrillation (OR = 3.867, 95% CI 1.970–7.591, *I*^2^ = 29.1%), NIHSS score (OR = 1.082, 95% CI 1.060–1.105, *I*^2^ = 54.5%) and onset-to-treatment time (OR = 1.003, 95% CI 1.001–1.005, *I*^2^ = 0.0%) were predictors of sICH after IVT. Alberta Stroke Program Early CT score (ASPECTS) (OR = 0.686, 95% CI 0.565–0.833, *I*^2^ =77.6%) and number of thrombectomy passes (OR = 1.374, 95% CI 1.012–1.866, *I*^2^ = 86.4%) were predictors of sICH after EVT.

**Conclusion:**

Several predictors of ICH were identified, which varied by treatment type. Studies based on larger and multi-center data sets should be prioritized to confirm the results.

**Systematic review registration:**

https://www.crd.york.ac.uk/prospero/display_record.php?RecordID=268927, identifier: CRD42021268927.

## 1. Introduction

Stroke is a leading cause of death and disability in Australia and around the world, with one in four people affected by stroke in their lifetime (1). Reperfusion therapies, including intravenous thrombolysis (IVT) and endovascular thrombectomy (EVT), can significantly improve patient outcomes (2) but are associated with complications, of which the most devastating is haemorrhagic transformation (HT). Haemorrhagic transformation after cerebral infarction is reported to occur in between 3.2 and 43.3% of strokes (3), and often results in a poorer prognosis. Aetiologically HT is a multifactorial phenomenon, and the ability to accurately predict the development of HT after reperfusion therapies has great potential to guide clinical decision making in order to maximize benefits and minimize harm.

Previous studies have identified many risk factors for HT, including (but not limited to) atrial fibrillation, higher baseline National Institute of Health Stroke Scale (NIHSS) score, advanced age, longer time from stroke onset to treatment (OTT), and lower baseline Alberta Stroke Program Early CT score (ASPECTS). Although a wide range of HT risk factors have been reported, findings have often been contradictory. For example, number of stent retriever passes at EVT has been variably reported to predict HT in comparable single-center cohorts (4–6), highlighting the heterogeneity of the evidence base.

Intravenous tissue plasminogen activator (tPA) improves outcome following ischaemic stroke when administered to appropriately selected patients up to 9 h after symptom onset (7–9). Endovascular thrombectomy (also known as mechanical thrombectomy), used either alone or in combination with IVT, has shown substantial benefit in patients with large vessel occlusion (10–14). Emerging evidence suggests that risk factors for HT vary considerably depending on the reperfusion treatment employed. In particular, higher rates of sICH have been reported following EVT (15), with certain imaging characteristics (occlusion site, ASPECTS) predicting HT in this setting (6, 16–19). Both individually and in combination, such predictors which are readily available in the hyperacute setting, have potential to guide clinical decision-making and prognostication.

This study reviews our current understanding of prognostic factors for HT in different treatment settings (IVT and EVT, respectively). Specifically, we aim to answer the review questions: (1) What are the baseline risk factors of haemorrhagic transformation after endovascular thrombectomy? (2) What are the baseline risk factors of haemorrhagic transformation after intravenous thrombolysis? (3) Is there any differences in risk factors of haemorrhagic transformation between endovascular thrombectomy and intravenous thrombolysis?

## 2. Methods

### 2.1. Search strategy

Electronic databases PubMed and EMBASE were used to identify relevant studies. The reference lists of eligible studies and systematic reviews were also checked and hand searching completed to find any additional relevant studies. The following search terms including their synonyms and available MeSH terms were used to retrieve relevant studies: Acute Ischemic Stroke, Hemorrhagic Transformation, Endovascular Thrombectomy, Intravenous Thrombolysis. The key search terms were combined using the Boolean operator “and” and “or” to retrieve the search results. Databases were searched from inception to August 2021.

### 2.2. Eligibility criteria

To be eligible for inclusion, studies were required to meet the following criteria: (1) Full-text publications in English. (2) Patients were diagnosed with acute ischaemic stroke. (3) Patient cohort aged 18 years old and over. (4) HT confirmed by CT/MRI scan within 48 hours after treatment. (5) Study included at least 50 patients. (6) Clinical or imaging data was measured prior to or during reperfusion treatment. (7) Treatment type of enrolled patients were either IVT or EVT, or bridging therapy (IVT plus EVT). (8) Predictors of HT were based on multivariate analysis and expressed as odds ratio (OR) with 95% CI.

### 2.3. Study screening and data extraction

Studies returned from the search results were screened using three steps. First, duplicate studies from across different databases were removed. Second, titles and abstracts of the search results were screened to check for eligibility by two independent reviewers (JS and CL), with disagreements resolved by discussion, and with a third reviewer (LC) if necessary. Finally, eligible full texts were screened by the same independent reviewers (JS and CL), with disagreements being resolved by discussion, and with a third reviewer if necessary.

For data extraction, two reviewers extracted the data independently using a predefined data extraction spreadsheet. Data were extracted from the selected studies guided by the CHARMS checklist (20), including authors and years, published journal, study type (randomized controlled trial or observational cohort), a single center or multi-center study, baseline characteristics of participants such as age, gender, onset-to-treatment time, NIHSS score, definitions of reported intracerebral hemorrhage (ICH) and the number of patients with HT, the HT confirmed timing after treatment, treatment type (intravenous therapy or endovascular therapy), risk factors identified and their type (continuous or categorized), regions and sample size. For performance measurements, odds ratio and 95% Confidence Interval (CI) and confounding variables adjusted in the multivariate analysis were extracted for prognostic factor studies.

### 2.4. Quality assessment

Two reviewers independently performed risk of bias assessments of the included studies. The two reviewers resolved any disagreements *via* discussion among themselves and with a third reviewer if required, until a consensus was reached.

To assess risk of bias in the included studies, the Quality In Prognosis Studies (QIPS) tool was used to evaluate validity and bias across six domains: participation, attrition, prognostic factor measurement, confounding measurement, and account, outcome measurement, and analysis and reporting (21).

### 2.5. Statistical analysis

Combined hemorrhagic transformation rates with 95% CIs were computed for symptomatic ICH (sICH) and any ICH, respectively. A meta-analysis of risk factors using extracted OR with 95% CI from individual studies was conducted if the risk factor was reported in a minimum of two studies. Odds ratio is an appropriate measure for categorical outcomes (22) and is a preferable report measure in meta-analysis on outcome prediction models (23). As well as odds ratio was the most prevalent measure reported in the included studies, we only extracted the odds ratio that was adjusted for confounding factors, which is preferable to analyses based on summary statistics according to Cochrane guidelines (24).

The *I*^2^ test was used to evaluate heterogeneity among included studies (25). For *I*^2^ statistic, 25, 50, and 75% were the threshold for low, moderate, and high heterogeneity. The τ^2^ was used to estimate the variance of the distribution of true effect sizes (26), and the confidence intervals around τ^2^ were calculated to quantify the uncertainty of heterogeneity (27). Prediction intervals were calculated to estimate the effect sizes of future studies based on present evidence (28). A random-effects model was used to analyse the data, regardless of heterogeneity. Begg's funnel plots were used to test potential publication bias for those results with number of studies > 10. Sensitivity analysis was conducted by removing included studies one by one to detect the influence of individual studies on the estimate of the overall effect. All statistical analyses were conducted with Stata software package (V.13.1; Stata, College Station, Texas, USA) and R 4.1.2 (R Foundation), with a *p*-value of *p* < 0.05 considered statistically significant.

## 3. Results

### 3.1. Literature search and study characteristics

**Figure 1:**
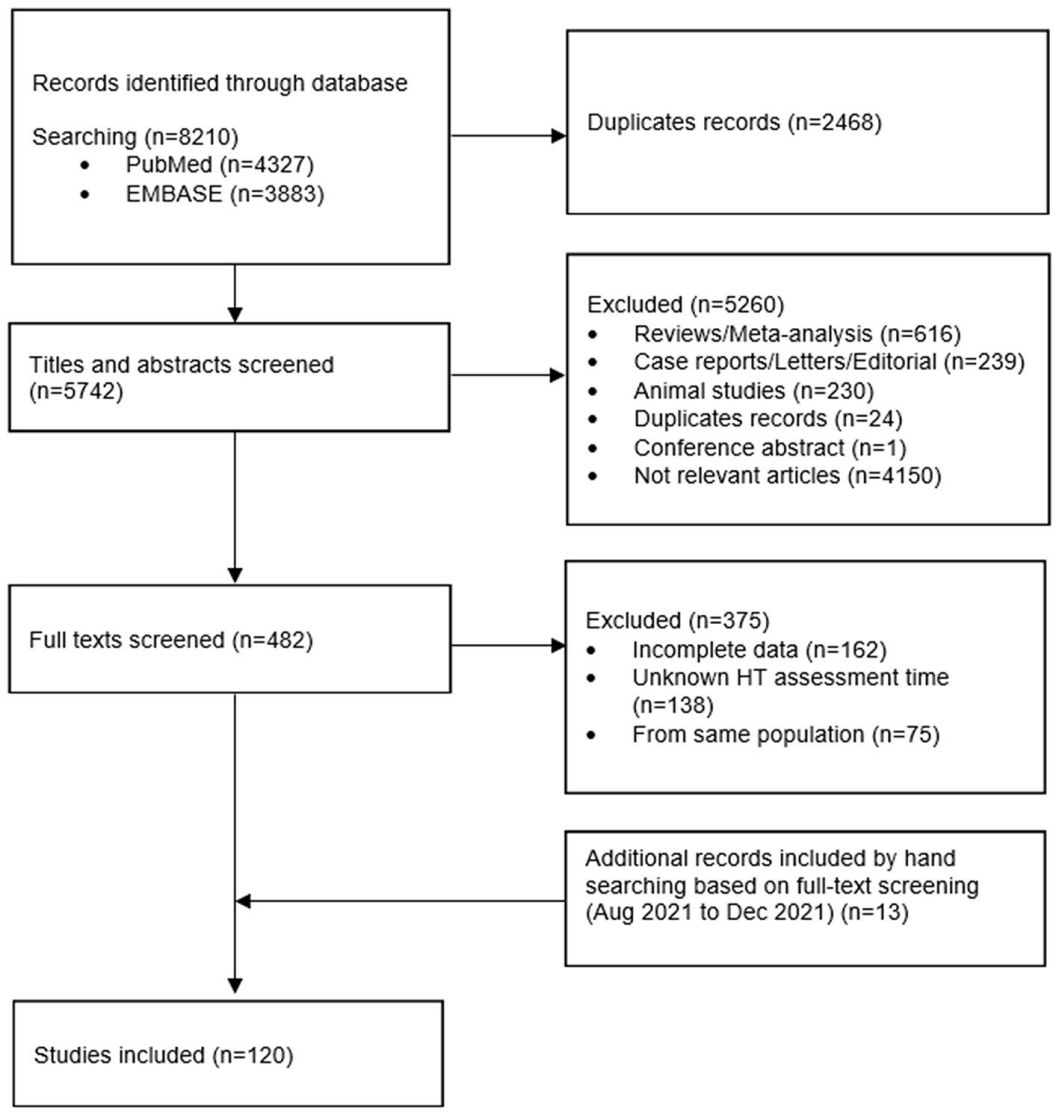
Screening flow diagram.

Literature search and screening processes are shown in Figure 1. Initially the search result included 5,742 articles after removing duplicates. After title and abstract screening, 482 articles remained. After full-text screening, 107 studies were included based on the search results, and another 13 relevant studies were identified *via* manual searching. In total, 120 studies (5, 6, 16–19, 29–142) were included in the meta-analysis.

Among the 120 included studies, 67 enrolled patients who were treated with IVT and 53 enrolled patients who were treated with EVT. Table 1 shows the characteristics of the included studies. The number of participants ranged from 71 (44) to 88,094 (55), with a total median sample size of 414 (Interquartile Range: 204.5–1,125). Further information on the characteristics of the included studies is summarized in Supplementary Table (“General characteristics”).

**Table 1:** Baseline characteristics of included studies.

	**Total**	**Treatment type**	
		**IVT**	**EVT**
Number of studies	120	67	53
Total sample size	345,477	300,979	44,498
Median sample size [range]	414 [204.5–1,125]	488 [235–1,475]	305 [199–751]

The general study quality was good, with a lack of reporting in the “Study confounding” domain in ~48% (58 out of 120) of the included studies. The results of quality assessment for each study are presented in Supplementary Table (“Quality assessment – QUIPS”) and Supplementary Figures 1, 2 (143).

### 3.2. Event rates of ICH and sICH

Tables 2, 3 show the event rates of any ICH and sICH per treatment type. In total, there were 32 IVT-based studies and 26 EVT-based studies that reported any ICH rates. Among the reported studies, any ICH rate ranged from 6.45% (106) to 49.55% (65), with a combined any ICH rate of 22.0% (95% CI 20.0–24.1%). The number of studies reporting sICH rates was 48 for IVT and 41 for EVT respectively. The sICH rate ranged from 1.27% (55) to 20.89% (129) with a combined sICH rate of 5.2% (95% CI 4.8–5.6%). Four main sICH criteria were applied in the included studies: the Safe Implementation of Thrombolysis in Stroke Monitoring Study (SITS-MOST) criteria (144), the European Cooperative Acute Stroke Study (ECASS) criteria (145), the National Institute of Neurological Diseases and Stroke (NINDS) criteria (7) and the Heidelberg Bleeding Classification (HBC) (146). The proportion of studies using each sICH criteria is shown in Supplementary Figure 3. In cases of multiple sICH criteria, SITS-MOST criteria were used to calculate the sICH rate; if SITS-MOST criteria were not reported, ECASS criteria were used.

**Table 2:** Any ICH rates per treatment type.

	**IVT**	**EVT**
Number of studies	32	26
Total sample size	48,657	13,615
Median sample size	369.5 [199, 681.5]	271 [187, 633]
Range of HT rates	6.45%-31.77%	7.60–49.55%
Combined HT rates 95% CI	15.3% (13.8–16.9%)	30.7% (26.4–34.9%)

**Table 3:** sICH rates per treatment type.

	**IVT**	**EVT**
Number of studies	48	41
Total sample size	263,470	36,824
Median sample size	818 [404.5, 2,173]	314 [205, 915]
Range of sICH rates	1.27–15.75%	1.52–20.89%
Combined sICH rates 95% CI	4.1% (3.7–4.5%)	7.2% (6.3–8.1%)

### 3.3. HT risk factors

In total, over 100 distinct risk factors were reported in the 113 prognostic factor studies. Since many risk factors were only reported in a single study, the meta-analysis included 24 risk factors that contributed to any ICH, and 32 risk factors that contributed to sICH. A summary of reported risk factors in the included study is shown in Supplementary Table (“Study results”).

### 3.4. Meta-analysis of risk factors related to ICH

**Figure 2:**
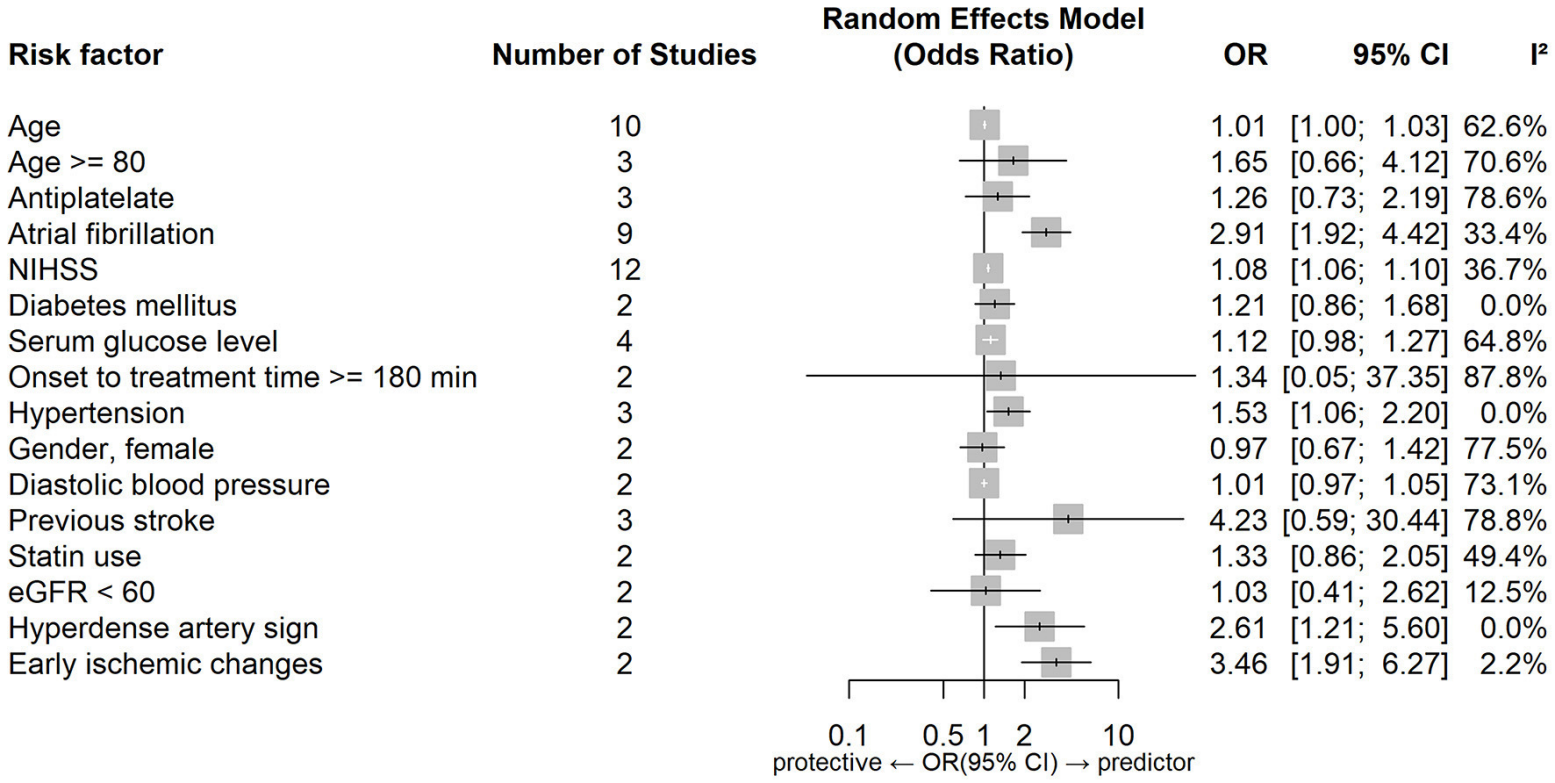
Forest plot of predictors for any ICH after IVT. OR, Odd Ratio; CI, Confidence Interval; NIHSS, National Institute of Health Stroke Scale; eGFR, estimated Glomerular Filtration Rate.

**Figure 3:**
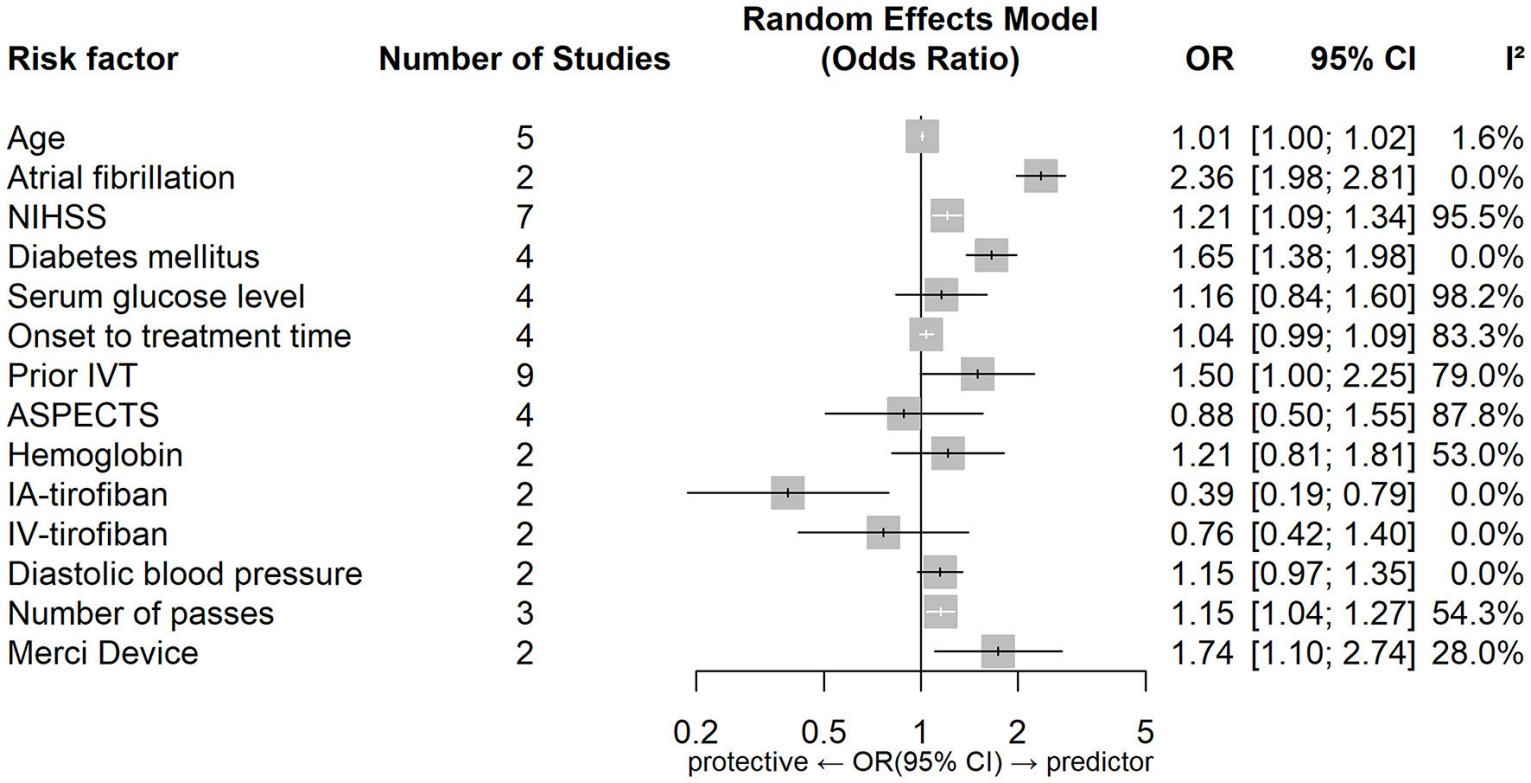
Forest plot of predictors for any ICH after EVT. OR, Odd Ratio; CI, Confidence Interval; NIHSS, National Institute of Health Stroke Scale; IVT, Intravenous Thrombolysis; ASPECTS, Alberta Stroke Program Early CT Score; IA, Intraarterial; IV, Intravenous.

Figures 2, 3 show forest plots of risk factors for any ICH (147). A combined total of 16 risk factors for any ICH after IVT and 14 risk factors for any ICH after EVT were included in the meta-analysis. Meta-analysis showed that early ischemic changes, atrial fibrillation, hyperdense artery sign, hypertension and NIHSS score were predictors for any ICH after IVT, while atrial fibrillation, use of the Merci Device, diabetes mellitus, NIHSS score and number of thrombectomy passes were predictors for any ICH after EVT. Intraarterial tirofiban was associated with a lower risk of any ICH after EVT. Table 4 lists predictors for any ICH.

**Table 4:** Predictors for any ICH in acute ischemic stroke patients: IVT vs. EVT.

**Risk factors**	**Treatment type**					
	**IVT**			**EVT**		
	**Number of studies**	**Combined OR (95% CI)**	*I* ^2^	**Number of studies**	**Combined OR (95% CI)**	*I* ^2^
Atrial fibrillation	9	2.912 (1.920–4.416)	33.4%	2	2.357 (1.978–2.809)	0.0%
NIHSS score	12	1.078 (1.058–1.099)	36.7%	7	1.208 (1.089–1.340)	95.5%
Diabetes mellitus	2	1.206 (0.865–1.683)	0.0%	4	1.655 (1.383–1.979)	0.0%
Hypertension	2	1.529 (1.060–2.205)	0.0%	N/A	N/A	N/A
Intraarterial tirofiban	N/A	N/A	N/A	2	0.386 (0.188–0.792)	0.0%
Number of passes	N/A	N/A	N/A	3	1.151 (1.041–1.272)	54.3%
Merci device	N/A	N/A	N/A	2	1.736 (1.101–2.739)	28.0%
Hyperdense artery sign	2	2.605 (1.212–5.599)	0.0%	N/A	N/A	N/A
Early ischemic changes	2	3.462 (1.912–6.268)	2.2%	N/A	N/A	N/A

### 3.5. Meta-analysis of risk factors related to sICH

**Figure 4:**
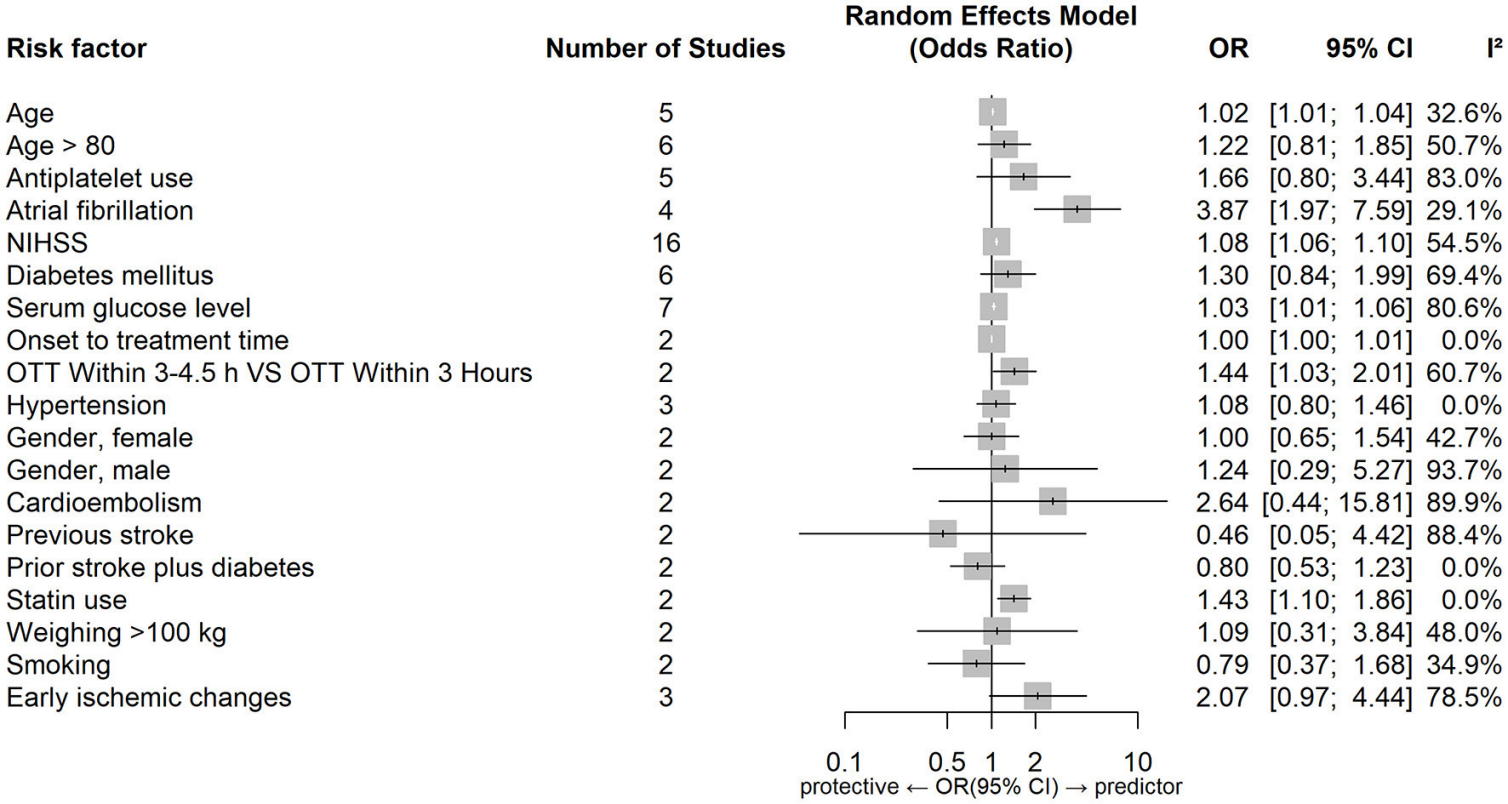
Forest plot of predictors for sICH after IVT. OR, Odd Ratio; CI, Confidence Interval; NIHSS, National Institute of Health Stroke Scale; OTT, Onset to Treatment Time.

**Figure 5:**
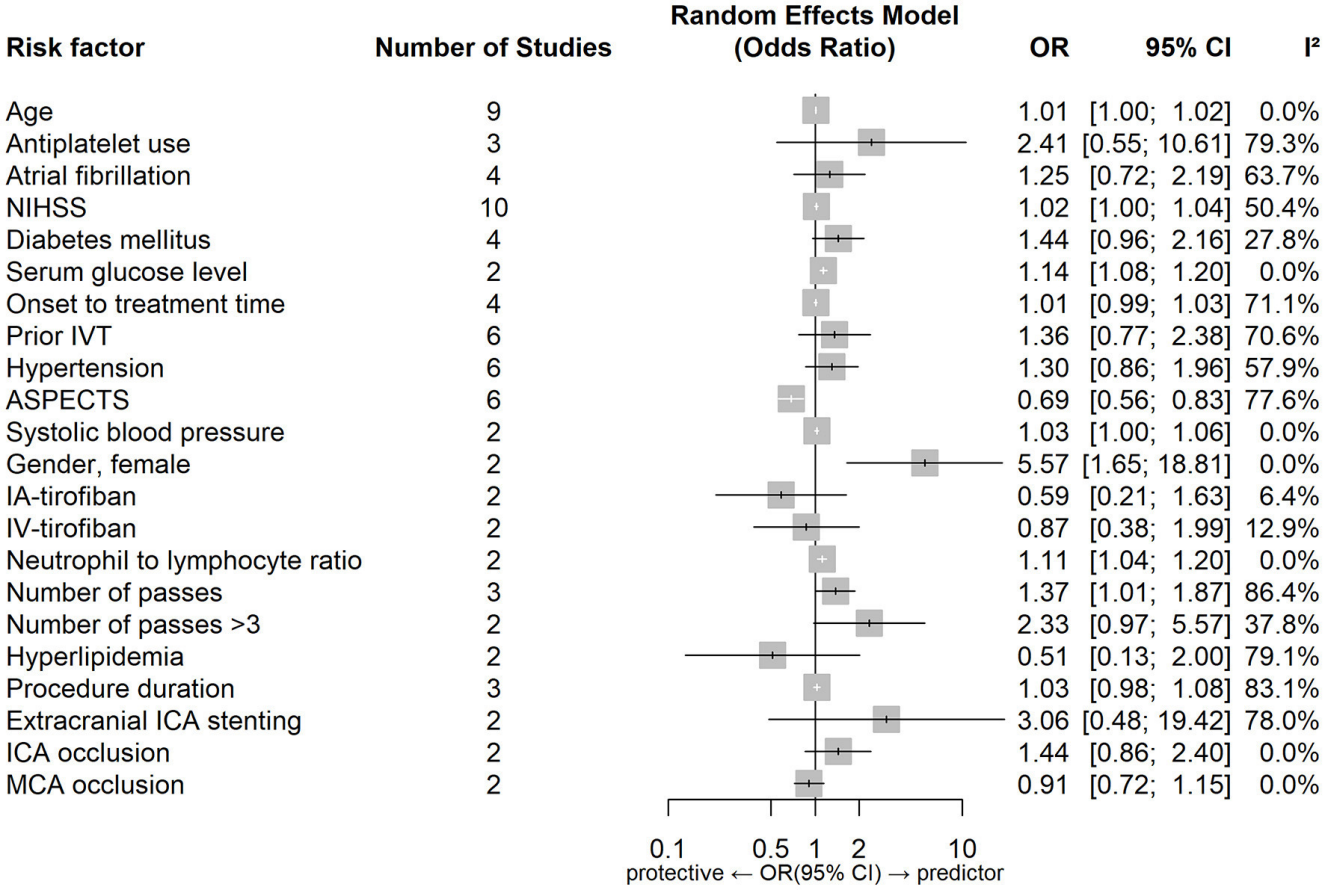
Forest plot of predictors for sICH after EVT. OR, Odd Ratio; CI, Confidence Interval; NIHSS, National Institute of Health Stroke Scale; IVT, Intravenous Thrombolysis; ASPECTS, Alberta Stroke Program Early CT Score; IA, Intraarterial; IV, Intravenous; ICA, Internal Carotid Artery; MCA, Middle Cerebral Artery.

Figures 4, 5 show forest plots of risk factors for sICH. A combined total of 19 risk factors for sICH after IVT and 22 risk factors for sICH after EVT were included in the meta-analysis. Meta-analysis showed that atrial fibrillation, OTT within 3–4.5 h VS OTT within 3 h, statin use, NIHSS score, serum glucose level, age and onset-to-treatment time were predictors of sICH after IVT, while female gender, number of thrombectomy passes, serum glucose level, neutrophil to lymphocyte ratio, age and lower ASPECTS were predictors of sICH after EVT. Table 5 lists predictors for sICH.

**Table 5:** Predictors for sICH in acute ischemic stroke patients: IVT vs. EVT.

**Risk factors**	**Treatment type**					
	**IVT**			**EVT**		
	**Number of studies**	**Combined OR (95% CI)**	*I* ^2^	**Number of studies**	**Combined OR (95% CI)**	*I* ^2^
Age (continuous)	5	1.023 (1.010–1.037)	32.6%	9	1.008 (1.001–1.016)	0.0%
Atrial fibrillation	4	3.867 (1.970–7.591)	29.1%	4	1.254 (0.719–2.185)	63.7%
NIHSS score	16	1.082 (1.060–1.105)	54.5%	10	1.019 (0.995–1.043)	50.4%
Serum glucose level	7	1.034 (1.009–1.060)	80.6%	2	1.141 (1.081–1.204)	0.0%
Onset-to-treatment time	2	1.003 (1.001–1.005)	0.0%	4	1.007 (0.987–1.027)	71.1%
OTT within 3–4.5 h vs. OTT within 3 h	2	1.437 (1.027–2.011)	60.7%	N/A	N/A	N/A
ASPECTS	N/A	N/A	N/A	6	0.686 (0.565–0.833)	77.6%
Gender, female	2	1.000 (0.649–1.541)	42.7%	2	5.568 (1.649–18.807)	0.0%
Neutrophil to lymphocyte ratio	N/A	N/A	N/A	2	1.114 (1.037–1.197)	0.0%
Number of passes	N/A	N/A	N/A	3	1.374 (1.012–1.866)	86.4%
Statin use	2	1.428 (1.097–1.858)	0.0%	N/A	N/A	N/A

### 3.6. Sensitivity analysis

In order to assess whether any particular study had a disproportionate influence on the meta-analysis results, heterogeneity assessment was done for the results with number of studies ≥3 and *I*^2^ ≥50%. For results where the confidence interval around τ^2^ did not contain zero, a further sensitivity analysis was done by removing one study at a time. Three results showed a statistically significant association between prior IVT and any ICH after EVT, but with very high heterogeneity (*I*^2^ ranged from 74–81%). The upper limit of the sensitivity analysis showed a statistically significant association between previous stroke and any ICH after IVT, with low-to-moderate heterogeneity but a very wide confidence interval (OR = 13.06, 95% CI 1.08–157.97, *I*^2^ = 46%). The upper limit of the sensitivity analysis also showed a statistically significant association between antiplatelet use and sICH after IVT with low heterogeneity (OR = 2.17, 95% CI 1.50–3.14, *I*^2^ = 0.0%), and also between antiplatelet use and sICH after EVT with high heterogeneity and a wide confidence interval (OR = 4.17, 95% CI 1.00–17.45, *I*^2^ = 83%). The upper limit of the sensitivity analysis also showed statistically significant association between early ischemic changes and sICH after IVT, with low to moderate heterogeneity (OR = 2.98, 95% CI 1.37–6.49, *I*^2^ = 44%). A summary of heterogeneity assessment and sensitivity analysis is shown in Supplementary Table (“Heterogeneity assessment” and “Sensitivity analysis”).

### 3.7. Assessment of publication bias

Funnel plots were used to assess publication bias for NIHSS score as a predictor of any ICH after IVT (number of studies *N* = 12) and sICH after IVT (number of studies *N* = 16). No evidence of publication bias was found for NIHSS score predicting any ICH after IVT (Egger's test, *P* = 0.185). However, there was evidence of significant small study bias for NIHSS score predicting sICH after IVT (Egger's test, intercept = 1.796, *t* = 4.48, *P* < 0.001), although one particular study (105) had a significant influence on this statistic.

## 4. Discussion

We performed a systematic review and meta-analysis to identify risk factors for HT after reperfusion therapies for acute ischaemic stroke. Although many factors have previously been reported as predictors of HT, findings are derived from widely varying studies.

### 4.1. Disparities in HT rates

The combined rates of both any ICH and sICH after EVT (30.7 and 7.2%) and IVT (15.3 and 4.1%) in our study were lower than those reported in previous work (35% for any ICH and 8% for sICH after EVT and 6.5% for sICH after IVT) (148, 149). As well as having a larger sample size, our analysis also standardized the definition of ICH across all included studies that reported multiple sICH criteria using SITS-MOST criteria. The incidence of HT was generally lower when these criteria were applied (150). In contrast, Hao et al. (148) study did not standardize the identification of ICH, and only 5% of their included studies used SITS-MOST criteria (vs. 18.4% in our study). Tsivgoulis et al. (149) review had a relatively small sample size (*N* = 12 vs. N ranging from 63 to 1,643 in other included studies), with only one study using SITS-MOST criteria.

Our analysis found that the combined rate of any ICH and sICH were lower in patients treated with IVT, in comparison to those treated with EVT. This result is consistent with previous reports (15). Several factors are thought to be responsible for higher HT rates after EVT. First, EVT studies by their nature include only patients with large vessel occlusion (LVO) stroke, who usually have worse stroke severity with higher NIHSS scores (151–153) and larger areas of involved tissue. Resulting ASPECTS scores are generally lower, and indeed this factor was found to independently predict sICH after EVT in our study. Second, in a finding corroborated by our analysis for the Merci device, the use of thrombectomy devices themselves have several implications for HT risk that center on vessel abrasion, including those arising from the type of device used and the number of passes required to achieve satisfactory reperfusion. Third, EVT is more commonly performed in patients with atrial fibrillation (18, 44, 96, 154) and with longer onset-to-treatment times (17, 68, 96), and uses additional antiplatelet agents during the procedure (112). Finally, other procedure-related factors such as the use of general anesthesia (155), distal embolization and extracranial stenting (16) may also contribute to a higher HT rate after EVT. These factors were not identified in our meta-analysis, due to a lack of relevant studies.

### 4.2. Predictors of any ICH

Common predictors for any ICH after reperfusion therapy (both IVT and EVT) were atrial fibrillation and a higher NIHSS score. While these two factors also predicted sICH after IVT they did not do so after EVT, however the relevant data set for EVT was limited by moderate-to-high heterogeneity. Hypertension, a hyperdense artery sign, and early ischemic change on non-contrast CT were predictors of any ICH after IVT and diabetes mellitus, number of thrombectomy passes and use of the Merci device were predictors of any ICH after EVT. Intriguingly, intraarterial tirofiban was found to be protective for any ICH after EVT.

Hyperdense artery sign and early ischemic changes were found to be predictors of any ICH after IVT. There were insufficient studies included in the meta-analysis that examined the same predictors for EVT. Hyperdense artery sign is associated with a higher clot burden and cardioembolic stroke, both of which predict a potentially larger area of infarcted brain tissue and a diminished response to thrombolysis (156–158). Early ischemic changes (including hypodensity and swelling/effacement) indicate the presence of brain oedema arising from prolonged hypoperfusion, and possibly the development of irreversible injury (159). These imaging features have been shown to predict HT, in particular where a significant portion (>33%) of the involved vascular territory is affected (160). Both hyperdense artery sign and early ischemic changes were imaging-based predictors derived from non-contrast CT, the most widely studied (and quantitative) stroke imaging modality.

Number of thrombectomy passes and use of the Merci device were predictors of any ICH after EVT, although the former result was subject to significant heterogeneity (prediction interval 0.4183–3.1667). Successive thrombectomy passes are thought to damage the arterial intima and weaken the vessel wall, causing micro-perforations at the time of device deployment/retraction (96) and so increasing the likelihood of HT (161). Use of the Merci device may increase vessel injury, vasospasm, or arterial dissection (96).

The effect of intraarterial tirofiban and intravenous tirofiban on HT risk varied in a key report (162), promoting us to regard route of administration of tirofiban as an independent variable. Surprisingly we found that intraarterial tirofiban was protective for any ICH after EVT. Among previous studies only Sun et al. (112) concluded that intraarterial tirofiban significantly decreased the odds of any ICH, with others either reporting contradictory or inconclusive findings (79, 163, 164). The contradictory findings may be explained by different rates of adjunctive IVT in these studies (increased ICH risks with adjunctive IVT). Sun et al. (162) study had a relatively small sample size (*N* = 195), and selection bias was introduced because use of tirofiban was administered at the neuro-interventional specialists' discretion. This is likely to have led to the exclusion of patients with larger infarct sizes who were at higher risk of subsequent ICH (165, 166). In addition, in patients receiving tirofiban it is possible that more stringent post-procedural blood pressure management may have been pursued, and the use of antiplatelet and anticoagulant therapies may have been more aggressively rationalized to reduce the risk of ICH (164). Notably Sun et al. conclusions were specific to patients with stroke due to large artery atherosclerosis, and did not reach significance for cardioembolism. Zhao et al. (164), who specifically recruited patients with cardioembolic stroke, concluded that intraarterial tirofiban was not protective for any ICH after EVT. Taken together these findings suggest that tirofiban's effect may be specific to both route of administration and stroke etiology.

### 4.3. Predictors of sICH

Common predictors for sICH after reperfusion therapy were higher age and a higher serum glucose level. Atrial fibrillation, a higher NIHSS score, longer onset-to-treatment time and statin use were predictors of sICH after IVT, while lower ASPECTS, female gender, higher neutrophil-to-lymphocyte ratio and number of thrombectomy passes were predictors of sICH after EVT.

Lower APSECTS (indicating larger stroke volumes) were found to independently predict sICH after EVT in our study. As described above a hyperglycaemic environment can impair cell metabolism and reduce vasoreactivity, which may disrupt the blood brain barrier integrity and increase the permeability, leading to the development of HT (5, 44, 101, 139, 167).

OTT within 3–4.5 h VS OTT within 3 h and statin use were found only to be the predictors of sICH after IVT, with insufficient studies to examine these associations for EVT. Although statin use was found to be predictive of sICH after IVT this finding was derived from two studies, with several others reporting no association between statin use and sICH (168–171).

Atrial fibrillation, a higher NIHSS score and longer onset-to-treatment time were found to be predictors of sICH after IVT but not after EVT. This result was also confirmed by the heterogeneity assessment, indicating there are different predictors of sICH after each treatment type.

A higher neutrophil-to-lymphocyte ratio was found only to predict sICH after EVT, there were insufficient results for IVT to examine the same factor. Neutrophil-to-lymphocyte ratio is a biomarker of systemic inflammation. Higher neutrophils lead to increased release of MMP-9 (matrix metalloproteinase-9) and disruption of neurovascular units and blood brain barrier integrity, increasing the risk of sICH (172–174).

### 4.4. Assessment of heterogeneity and publication bias

In this study, heterogeneity assessment and sensitivity analysis were done to explore the robustness of the results. Heterogeneity assessment was performed for 25 results and a further sensitivity analysis was done for 11 results, of which six differed from the original findings. The robustness of the meta-analysis was therefore generally good.

The between-study heterogeneity observed could be explained by several factors. First, different study designs with different inclusion criteria resulted in large variations in patient characteristics including stroke severity, average age, onset-to-treatment time, disease history and stroke etiology. There was also significant variation in the number and the ethnicity of enrolled patients. Second, definitions of the same risk factors were not standardized across different studies, and in some studies were ambiguous or not clearly stated. For example, there was no uniform method of defining hyperdense artery sign: a clot with a Hounsfield unit ratio of 1.1 indicated a hyperdense artery sign in one study (175), while in another a ratio of 1.5 was used to exclude a hyperdense artery sign (176). Furthermore, for drug related risk factors, different studies used different medication regimes with regard to type, dose, and timing of administration. For example, while prior antiplatelet use was identified as a predictor in multiple studies, the specific drug varied despite the fact that different agents are recognized to variably affect HT (177). Previous work has also demonstrated that different doses of tirofiban could have different effects on HT (163). Third, measurement bias, especially for biomarkers, is likely to have been a factor. A previous review reported that the definition of hyperglycaemia varied from study to study, and the measurement methods used included both random and fasting serum levels (178). Fourth, as previously mentioned, four different kinds of sICH criteria were used across the included studies, which is likely to have caused the rates of sICH identified to vary significantly. Lastly, studies went to different lengths to adjust for confounders in their multivariate analyses, or made no adjustments at all.

Finally, one of our two assessments for publication bias (NIHSS score predicting sICH after IVT) showed significant evidence of small study bias, possibly because we did not include abstracts or search the gray literature. As a result, some negative studies may have been omitted. However, the asymmetry of the funnel plots can also be caused by between-study heterogeneity (179), and heterogeneity assessment showed that the result of NIHSS score predicting sICH after IVT had moderate to high heterogeneity (*I*^2^ = 54.5%).

### 4.5. Strengths and limitations

This study is the first to compare risk factors for HT following different treatment types (IVT vs. EVT), an approach which has the potential to guide patient selection and clinical decision-making. It is also the first study to systematically review risk factors for sICH after IVT and the second to systematically review risk factors of HT after EVT (148). Although several systematic reviews have examined risk factors for HT after IVT (165, 180, 181), they did not differentiate “any ICH” from sICH. Several studies were also limited by the use of geographically restricted (often Chinese) patient groups. Compared to any ICH, sICH is more likely to predict a poor prognosis, making any study that identifies predictors of sICH of particular clinical relevance.

Our study has several limitations. First, we were unable to conduct a meta-analysis incorporating all reported risk factors because many were only reported in single studies. We also did not analyze risk factors for HT based on radiological criteria (hemorrhagic infarction and parenchymal hematoma) due to limited studies reported relevant information in our included studies. However, a systematic review (182) investigating predictors for different radiological degrees of HT had similar findings with this study. Second, there were large disparities in HT rates (6.45 to 49.55% in any ICH), indicating substantial differences of methodology across different incorporated studies. This possibly renders the meta-analysis results were very unstable and questions whether the pooled studies were homogenous. Although we performed heterogeneity assessment and sensitivity analysis to examine the robustness of the meta-analysis results, the meta-analysis results still need to be interpreted carefully. Third, we did not account for measures made to minimize the risk and extent of HT such as tight blood pressure and glucose control post procedure. Fourth, we did not differentiate treatment type of thrombectomy only and bridging therapy when analyzing the combined HT rates and predictors for HT. Fifth, we did not perform subgroup analysis by removing studies with a high risk of bias. However, results of sensitivity analysis demonstrated the general robustness of the meta-analysis findings and identified specific finding that needed to be interpreted with caution. Sixth, we only searched two electronic databases for the literature search. Nevertheless, initial search results returned nearly six thousand non-duplicated studies and additional manual searching was done to mitigate this potential risk of bias. Lastly, we did not search the gray (unpublished) literature in order to mitigate the risk of publication bias.

### 4.6. Implications for clinical practice

When treating patients with EVT, neuro-interventionalists should consider the impact of multiple retrieval attempts/device passes and be mindful of their choice of thrombectomy device, as both were found to be predictors of any ICH in this study. In addition, patients with atrial fibrillation, a higher NIHSS score, higher age, or a higher serum glucose level should be considered in the highest risk category for HT in the hours and stays after hyperacute management, regardless of treatment.

### 4.7. Recommendations for future research

EVT studies are generally more recent than IVT studies (respectively 64.2 and 20.9% were published after 2020). EVT studies are also fewer in number (*N* = 53 vs. *N* = 67) and are generally smaller in size (median sample size 305 compared to 488). Meanwhile 27 studies proposed imaging-based predictors for HT, with 20 published after 2018. Improved imaging technology in recent years, particularly the advent of CT perfusion, have shown great potential to enhance patient selection by more accurately characterizing infarct core and penumbra. For example, CT perfusion-based predictors including those measuring infarct core volume (19, 53, 183, 184) and blood-brain-barrier permeability (6, 142, 185–188) haven been reported in previous work, and novel CT perfusion-based parameters such as net water uptake (189) continue to emerge. These techniques have largely emerged in tandem with EVT and indeed have enabled its application in the extended therapeutic window, meaning that the majority of published randomized trial data charactering HT has EVT as a focus. Conversely, data for IVT is in the main derived from trials using CT/CTA [although CTP-directed thrombolysis is an emerging evidence-based treatment approach (190)]. Furthermore, use of Tenecteplase, a newer thrombolytic agent with improved ease of use and a potentially more favorable safety profile, has been less widely studied. Core areas for future studies therefore include (a) novel imaging predictors of HT (particularly those using CT perfusion, given its use in the hyperacute setting) and (b) HT rates/characteristics after the administration of Tenecteplase (with and without EVT).

Both the use of multiple criteria for sICH and substantial variation in the timing of follow-up imaging introduced significant heterogeneity into the meta-analysis. Future studies should be harmonized to incorporate the use of SITS-MOST criteria to characterize HT in scans not performed more than 48 h after hyperacute therapy. These two simple steps would ameliorate much of the variability we found.

## 5. Conclusion

Hemorrhagic transformation is one of the most devastating complications of reperfusion therapy for patients with acute ischaemic stroke. This meta-analysis identified several predictors for HT, including atrial fibrillation, a higher NIHSS score, higher age, a higher serum glucose level number of thrombectomy passes, and lower ASPECTS. Key predictors for HT in the published literature, identified here, will form the basis for future studies. However, given the large disparities and heterogeneity across the included studies, the meta-analysis results need to be interpreted with caution, and studies based on larger and multi-center data sets should be prioritized to confirm the results.

## Data availability statement

The original contributions presented in the study are included in the article/Supplementary material, further inquiries can be directed to the corresponding authors.

## Author contributions

JS and CL screened abstracts and titles of potentially relevant studies, screened the full-text papers, and extracted data and assessed the quality independently. JS performed all statistical analysis, drafted the manuscript, and made critical revisions to the manuscript. LC contributed to the design of the study and made critical revisions to the manuscript. MP and AB conceived the study and made critical revisions to the manuscript. LL, CB, and XL critically reviewed the manuscript. All authors contributed to the article and approved the submitted version.

## Supplementary material

Supplementary Figure 1 Risk of bias QUIPS summary.

Supplementary Figure 2 Risk of bias QUIPS traffic light.

Supplementary Figure 3 The proportions of sICH definitions in included studies.
