# Supplementary figures and images for: Risk factors of hemorrhagic transformation in acute ischaemic stroke: A systematic review and meta-analysis
Source: Front Neurol. 2023 Feb 20;14:1079205. doi: 10.3389/fneur.2023.1079205 (PMC9986457; doi:10.3389/fneur.2023.1079205)

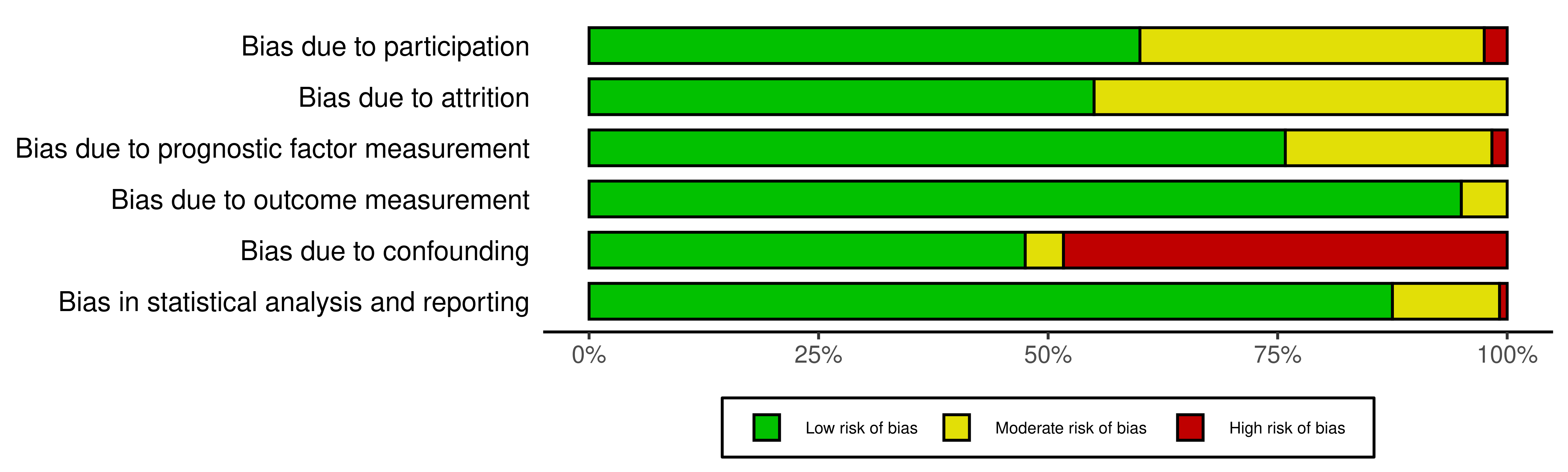

Supplement: Supplementary Figure 1 — Risk of bias QUIPS summary. [file Image_1.PNG]

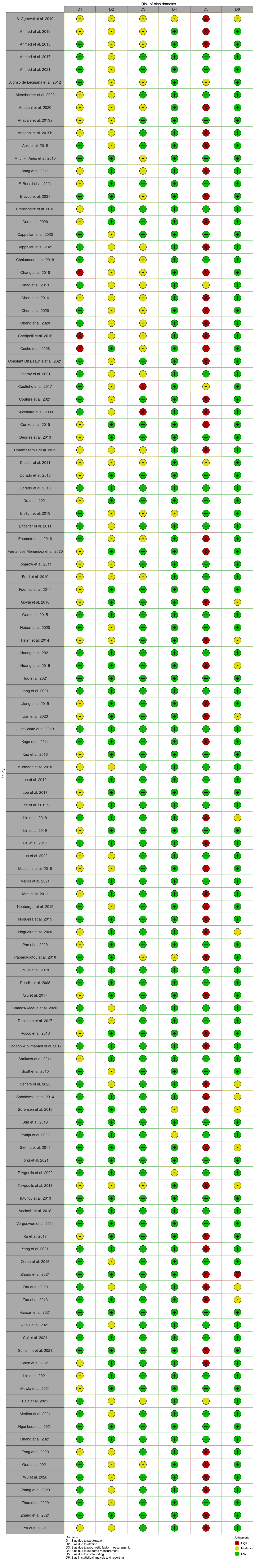

Supplement: Supplementary Figure 2 — Risk of bias QUIPS traffic light. [file Image_2.PNG]

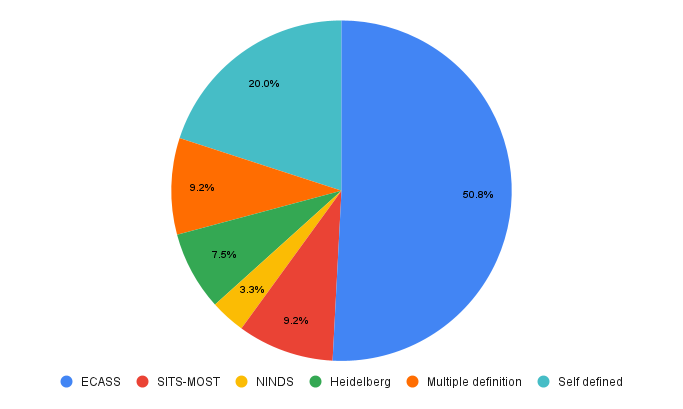

Supplement: Supplementary Figure 3 — The proportions of sICH definitions in included studies. [file Image_3.PNG]
